# Acute external iliac artery thrombosis following pelvic fractures: Two case reports

**DOI:** 10.1097/MD.0000000000024710

**Published:** 2021-02-12

**Authors:** Shuliang Zhang, Hongfeng Sheng, Bin Xu, Yangjun Lao

**Affiliations:** Department of Orthopaedics, Tongde Hospital of Zhejiang Province, Hangzhou, China.

**Keywords:** case report, external iliac artery, pelvic fractures, thrombosis

## Abstract

**Rationale:**

Pelvic fractures associated with acute external iliac artery thrombosis is less common, it is easily ignored in clinical practice, and it can result in limb amputation and hemipelvectomy due to prolonged limb ischemia. We present 2 patients with acute external iliac artery thrombosis following pelvic fractures.

**Patient concerns:**

Case 1 is a 49-year-old male with occlusion of the right external iliac artery and pelvic fractures. Case 2 is a 52-year-old male with left external iliac artery occlusion and bilateral internal iliac artery rupture following pelvic fractures.

**Diagnoses:**

Case 1 was diagnosed with computed tomography angiography. Case 2 was diagnosed with ultrasound examination and computed tomography angiography.

**Interventions:**

Case 1 was performed an open incision to remove thrombus of the right external iliac artery, the right iliac-femoral artery artificial bypass was adopted to restore the blood flow. Case 2 underwent segmental resection of the damaged artery and artificial vascular implantation of left external iliac artery, and angiographic embolization of bilateral internal iliac artery. However, a left hip disarticulation was performed due to osteofascial compartment syndrome at last.

**Outcomes:**

Case 1 was cured and discharged smoothly. Case 2 survived but left with a disability after disarticulation.

**Lessons:**

Acute external iliac artery thrombosis after pelvic fractures is rare and limb-threatening, life-threatening. It is very important to detect and treat this potential complication timely when a patient with a pelvic fracture.

## 1 Introduction

The prevalence of pelvic fractures is more often associated with high energy injury, it leads to high mortality, the reported mortality rate is 18% to 40%,^[1]^ and the major cause of death of pelvic fractures patients is massive bleeding.^[2]^ Even low-energy pelvic fractures can lead to life-threatening hemorrhage.^[3]^ As a result, doctors pay more attention to the hemodynamic stability of pelvic fractures patients prehospital and after admission. The French Society of Anaesthesia and Intensive Care Medicine and the French Society of Emergency Medicine issued 1 guideline suggested that the management of pelvic fractures in the first 24 hours focuses on the finding of bleeding point and the control of bleeding.^[4]^ Moreover, the occlusion of a major pelvic artery is rare, so the arterial thrombosis of pelvic fractures patients may be ignored. But when this occurs, it needs extensive debridement, even limb amputation and hemipelvectomy are finally performed due to prolonged limb ischemia and tissue necrosis.^[5]^

Because of the retroperitoneal location of the iliac vessels, pelvic fractures associated with external iliac artery injury and occlusion is less common, with only a few sporadic cases reported.^[6–10]^ In the past year, 13 cases with pelvic fractures were admitted to our ward, of which 2 cases developed external iliac artery thrombosis within 24 hours. This article reports the 2 cases and shares our experience. This study was approved by the Ethics Committee of Tongde Hospital of Zhejiang Province. Written informed consent was obtained from the patients for publication of the 2 case reports and accompanying images.

## 2 Case reports

### 2.1 Case 1

**Figure 1:**
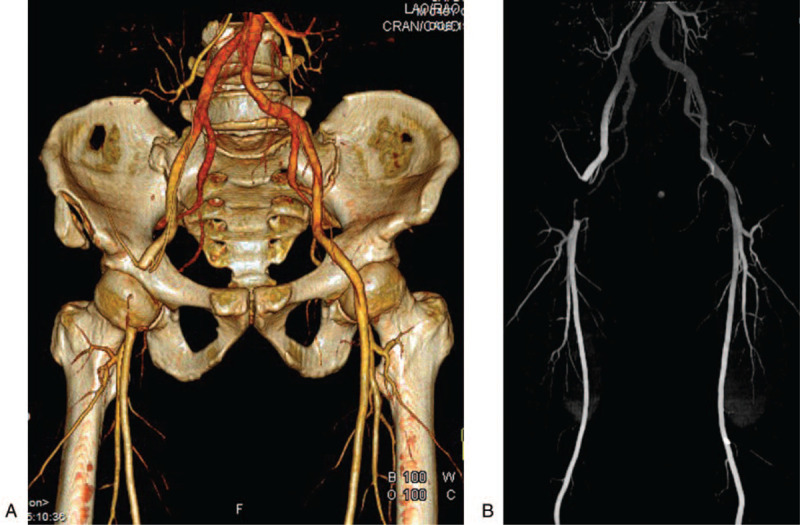
Case 1, CTA showed the right ilium wing fracture and a total occlusion of the distal end of the right external iliac artery. CTA = computed tomography angiography.

A 49-year-old male fell from a height and arrived at the emergency department of our hospital half an hour after the accident. X-ray was performed at once and revealed a fracture of the right ilium wing. He entered the inpatient department 1 hour later. On physical examination, the dorsal foot artery pulses in both lower extremities were normal. His vital signs were stable with 66 beats per minute of heart rate, 126/85 mm Hg of blood pressure, 18 breaths per minute of respiratory rate. He has stable hemodynamics. 15 hours later, physical examination was performed once again and found that the patient had no palpable pulses in the right lower extremity, the skin temperature was lower than that of the left lower limb. An emergent pelvic and lower extremity computed tomography angiography (CTA) revealed a total occlusion of the distal end of the right external iliac artery (Fig. 1).

The patient was taken into the operating room and given general anesthesia. At first, the percutaneous intervention treatment was implemented. However, repeated operation of the guidewire failed to pass through the diseased segments of the right external iliac artery. Finally, an incision was performed to open up the artery, when the thrombus was removed, the right iliac-femoral artery artificial bypass was performed. During the operation, a contused right external iliac artery was demonstrated. At the end of the procedure, the right lower limb had a strong pulse, the skin of the right lower limb warmed again. On the second day review, CTA showed clear blood flow in the right external iliac artery. One week later, the fracture of the right ilium wing was fixed with 2 hollow screws.

### 2.2 Case 2

**Figure 2:**
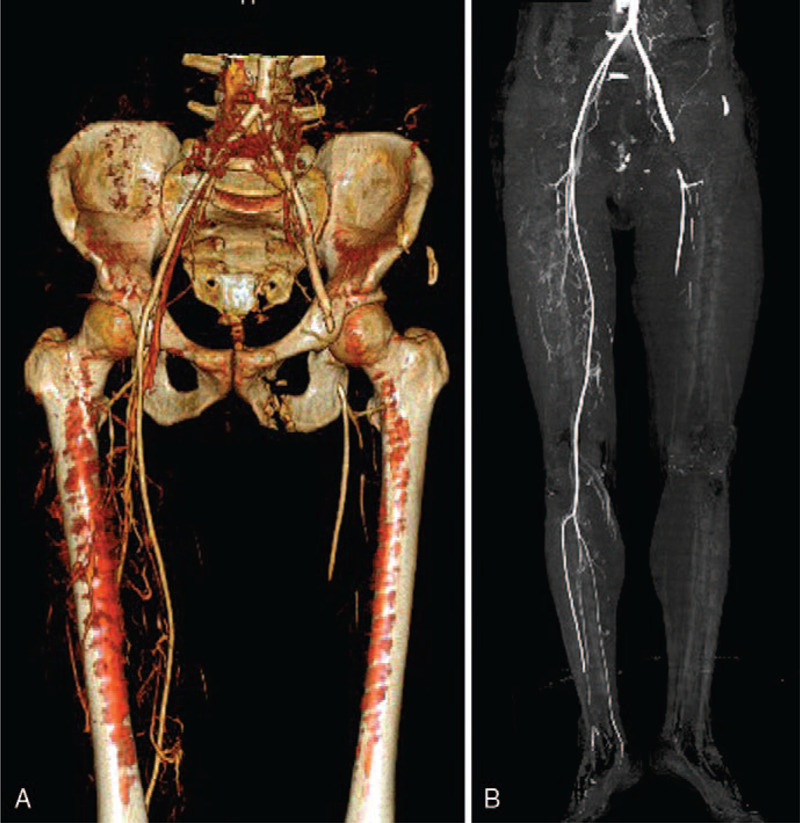
Case 2, CTA showed multiple fractures of the pelvic and a total occlusion of the distal end of the left external iliac artery and the left femoral artery. CTA = computed tomography angiography.

A 52-year-old male was hit by a car while walking down the road. He had a headache, dizziness, no coma, or loss of consciousness at the time. He presented to the emergency department with severe pain in his lower abdomen and left thigh. He was hemodynamically stable, initial BP was 132/82 mm Hg and P was 72/minutes. His initial physical examination revealed positive pelvic compression and separation test, tenderness in the left ventral trigonum, obvious swelling of the left thigh. Initial computed tomographic (CT) imaging showed multiple fractures of the sacrum, bilateral ilium, left pubic bone and acetabulum, and no intracranial abnormalities. One hour later, he entered the inpatient department. Physical examination was performed once again by the resident. It presented a reduction of power and numbness in his left thigh which was cold and pale, weakened pulses of the left dorsal foot artery, bloody fluid flowing out of the urethral opening. His hemodynamics became unstable. His blood pressure fluctuates between 90 to 120 and 50 to 70 mm Hg. Ultrasound examination was performed immediately, it indicated that the left common femoral artery had poor blood flow and no obvious blood flow signal was detected in the left dorsal foot artery. Further CTA examination revealed occlusion of the distal end of the left external iliac artery and the left femoral artery, inferior artery of them was not seen, and exudative shadows were seen in the pelvic cavity and retroperitoneum, and circular high-density shadows were seen around the bladder. Thus, the diagnoses of pelvic hemorrhage, arterial thrombosis, urethral injury were obtained (Fig. 2).

The patient was admitted to the emergency operating room. The urologist diagnosed the patient was a urethral tear and failed to catheterize, the patient underwent vesical puncture ostomy. Next procedure, a pelvic external fïxator was applied. During the operation, extravasation of contrast agent from bilateral internal iliac artery was found, there was bleeding from the ruptured internal iliac artery. As a result, angiographic embolization was performed for the control of pelvis bleeding. Then, an 8 cm incision was made in the left inguinal area. Thromboembolism was detected in the distal end of the left external iliac artery and the left femoral artery. The arteries and the surrounding muscles were severely damaged and the arteries’ pulse disappeared. The severely damaged section of the arteries was resected, and the artificial blood vessel was implanted, and then the arterial pulse was restored. A second intraoperative angiogram showed clear blood flow of the iliac-femoral artery and the artificial vessels, occlusion of the left popliteal artery. Through the interventional method, many thrombi were removed after repeated 3 times of thrombectomy from the left popliteal artery. The third intraoperative angiography revealed patency of the left popliteal artery, left anterior tibial artery, left posterior tibial artery, and peroneal artery. The patient was transferred into the intensive care unit (ICU) after surgery.

About 10 hours later, swelling of the left lower limb was aggravated. Osteofascial compartment syndrome was considered and the second operation was adopted. The detection found that the muscles of the left thigh, left calf and left plantar were bright red, but their contractility and elasticity was reduced. After the operation of intraosseous compartment incision and decompression, the popliteal artery and anterior tibial artery pulsation were palpable, and the terminal blood circulation was better than before. The patient was transferred back to ICU for further treatment. Five days later, the third operation was performed. There was complete necrosis of the left vastus lateralis muscle, inactivation and necrosis of the medial and lateral muscles of the left calf. A thorough debridement was applied. Another 5 days later, the patient had a persisting fever, the indicators of infection increased, the indicators of liver and kidney function worsened, and the symptoms of systemic poisoning were obvious, multiple organ dysfunction occurred. The fourth operation of debridement was performed. Intraoperatively, necrosis muscles of the entire left thigh and the left calf was observed. Therefore, a left hip disarticulation was performed to ensure the patient's survival.

## 3 Discussion

The pelvic fracture itself is not a fatal fracture, its high mortality rate is primarily contributed by massive intra-pelvic bleeding. Bleeding associated with pelvic fractures mainly comes from the disruption of the presacral and lumbar venous plexus, or directly from the fracture site.^[5]^ Major artery injury secondary to pelvic fractures is rare. It is reported that the incidence of iliac artery injuries ranges from 0.4% to 7.1%.^[11]^ Arterial bleeding of pelvic fractures occurs commonly from the internal iliac artery.^[12]^ In 1 study, the patients with pelvic trauma who underwent angiography demonstrated that 97% of artery injury happened to the branches of the internal iliac artery.^[13]^ The injury of the external iliac artery is more likely followed by thrombosis,^[14]^ but thrombosis originating from the lesion of the external iliac artery is extremely uncommon. There is no large-scale cases had been reported, but only a few sporadic cases were issued.^[6–10]^ In this study, case 1 suffered the right external iliac artery thrombosis, case 2 suffered left external iliac artery thrombosis and bilateral internal iliac artery rupture hemorrhage. To our knowledge, there have been no previous reports of simultaneous internal iliac artery bleeding and external iliac artery thrombosis like case 2.

The iliac artery injury can result from several mechanisms of blunt trauma and include direct impact injury, shear injury, direct compression injury.^[12]^ The enormous force from the anterolateral area acts directly on the iliac wing causing damage to the iliac artery, this direct impact injury can happen in traffic accidents and falling injuries. The iliac artery has some certain connections with the pelvic wall through a few ligaments and fascias. During the traumatic event, intimal tears, or atherosclerotic plaque rupture as a result of tensile stresses of ligament and fascia can occur.^[15]^ The rapid deceleration in traffic collisions produces shearing forces, which lead to longitudinal stretching of the vessels, the vessel wall will be damaged.^[12]^ Direct compression on the vessels often happens in seatbelt injury, or when the vessels squeezed by 2 fracture blocks. Otherwise, the sharp broken bone end can directly cut the external iliac artery, or the patient can pierce the external iliac artery with the fracture block in the later transport process.^[16]^ All the above injuries can cause partial-thickness or full-thickness damage to the intima of the vessels, platelet will aggregate form at the damaged site of the intima, resulting in partial or complete thrombosis of a vessel lumen. It is can be demonstrated by our cases. The right external iliac artery intima of case 1 was badly contused, and the left external iliac artery intima of case 2 was severely damaged.

Arterial thrombosis is often not given enough attention in the clinical practice. In the pelvic fracture patients with artery injury, control of bleeding always takes priority. The arterial thrombosis of the pelvic fracture patients could be ignored. As a result, when the injury of the external iliac artery occurs, it usually results in death or disability.^[17]^ Lower extremity amputation was 5 times as common and compartment syndrome was almost 3 times more likely when the external iliac artery thrombosis was present. Patients with external iliac artery thrombosis were more than twice as likely to die as those with other vascular injuries.^[17]^ Raffaele reported that there was only 5 patients had external iliac artery lesion among the 281 pelvic fractures, the incidence was 1.7%,^[5]^ 2 died before angiographic. Of the 3 patients who had thrombosed artery, 2 underwent urgent hemipelvectomy, and 1 died. Harris^[6]^ found that the amputation rate was high for the people with the external iliac artery injury, 67% of these people who underwent amputation required a hemipelvectomy. Even though 80% of the people who had undergone revascularization had to required amputation at last. Cestero^[17]^ and Carillo^[14]^ reported amputation rates of 8% and 25%, respectively. In this study, case 2 underwent a left hip disarticulation.

The patients with the external iliac artery injury mainly present with signs of leg malperfusion, such as pulseless, cold, cyanotic, impaired motor and sensory functions, numbness, and tingling in the leg and foot.^[8]^ Once these clinical manifestations occur in patients with pelvic fractures, the possibility of external iliac artery thrombosis should be considered immediately. An angiographic examination must be performed immediately, and emergent intervention must be taken if found. If arterial thrombosis is not detected in time, it can lead to ischemia and necrosis of the lower extremity muscles, resulting in amputation or death. Carillo^[14]^ reported 1 patient underwent amputation because his left external iliac thrombosis was undiagnosed while the patient underwent abdominal surgery for multiple associated injuries. Smejkal^[10]^ reported 1 patient who underwent a right hip disarticulation following occluded of the right external iliac artery. He died of multiple organ failure caused by sepsis and myonecrosis. Our 2 cases appeared pulseless, cold, reduction of power, and numbness within 24 hours of admission, we performed prompt angiographic examination and surgery, but case 2 still regretfully underwent amputation.

Thrombolysis^[18]^ and surgery are the 2 methods for the management of arterial thrombosis. Surgical techniques include interventional thrombectomy and endovascular stenting, open thrombectomy followed by end-to-end anastomosis, reconstruction with prosthetic conduit, or with great saphenous vein graft, an anatomic or extra-anatomic bypass procedure as well as reconstructions are valuable choice.^[19]^ In this study, it was impossible to choose thrombolysis for the 2 patients, because 2 patients had the pelvic fracture bleeding, and case 2 had internal iliac artery rupture and bleeding. Finally, the 2 cases were treated with surgery. Case 1 expressed satisfaction with the emergency treatment we took, as these treatments saved his leg. Due to the severe high-energy trauma, case 2 was grateful for our treatment to save his life.

In conclusion, acute occlusion of the external iliac artery is a rare and severe complication of patients with pelvic fractures, which can be life-threatening. It is very important to detect and treat this potential complication timely when a patient with a pelvic fracture.

## Acknowledgments

The authors are grateful to the 2 patients.

## Author contributions

**Conceptualization:** Shuliang Zhang.

**Formal analysis:** Shuliang Zhang, Hongfeng Sheng, Yangjun Lao.

**Investigation:** Shuliang Zhang, Hongfeng Sheng.

**Project administration:** Hongfeng Sheng, Bin Xu, Yangjun Lao.

**Resources:** Shuliang Zhang, Hongfeng Sheng.

**Writing – original draft:** Shuliang Zhang.

**Writing – review & editing:** Shuliang Zhang, Hongfeng Sheng.

## Abbreviations

Abbreviations: CT = computed tomographic, CTA = computed tomography angiography, ICU = intensive care unit.
